# Transcriptional activation of Proteasome 26S non-ATPase subunit 7 by forkhead box P3 participates in gastric cancer cell proliferation and apoptosis

**DOI:** 10.1080/21655979.2021.2018097

**Published:** 2022-01-17

**Authors:** Yujie Xu, Dingmao Wang, Guodong Zhao

**Affiliations:** Department of Gastrointestinal Surgery, Haikou People’s Hospital, Haikou, Hainan Province, China

**Keywords:** PSMD7, FOXP3, gastric cancer, transcriptional activation, apoptosis

## Abstract

Proteasome 26S non-ATPase subunit 7 (PSMD7) and forkhead box P3 (FOXP3) have been found to be both upregulated in gastric cancer tissues. FOXP3 was also predicted to have binding sites on PSMD7 promoter. Thus, this study investigated the relationship between PSMD7 and FOXP3 and their roles in gastric cancer. Bioinformatic databases predicted PSMD7 expression in non-cancerous gastric tissue and gastric cancer tissue, as well as the correlation between PSMD7 and the overall/disease free survival. PSMD7 expression in non-cancerous gastric tissue or cells and gastric cancer tissue or cells was detected by qPCR and Western blot. After PSMD7 downregulation by siRNA interference, cell viability, colony-forming capacity and cell apoptosis were analyzed with cell counting kit-8 assay, colony formation assay and terminal deoxynucleotidyl transferasemediated dUTP nick end-labeling. Proliferation and apoptosis markers were assayed by qPCR and Western blot. Dual-luciferase reporter and chromatin immunoprecipitation assays were performed to look at the binding relationship between FOXP3 and PSMD7 promoter. Cell proliferation and apoptosis were examined again after co-transfection of PSMD7 siRNA plasmid and FOXP3 overexpression plasmid. PSMD7 expression was much higher in gastric cancer tissue and cell lines. Interference with PSMD7 decreased gastric cancer cell viability, inhibited their proliferation and colony formation and promoted cell apoptosis. FOXP3 was found to bind to PSMD7 promoter and activate PSMD7 expression. Overexpression of FOXP3 could rescue the effects of PSMD7 knockdown on gastric cancer cells. PSMD7 is involved in the proliferation and apoptosis of gastric cancer cells and can be transcriptionally regulated by FOXP3.

## Introduction

Gastric cancer, cancer of the stomach, is originally derived from gastric mucosal epithelial cells [1]. The five-year survival rate of early gastric cancer after surgery can reach 90%, but there is a lack of effective treatment for advanced gastric cancer [2]. Even with active combined therapies, the five-year survival rate of advanced gastric cancer is still less than 30% [3,4]. Adenocarcinoma is the most common pathological type of this malignancy [5]. In most cases, by the time of the diagnosis of gastric cancer, the cancerous tissue has diffused and infiltrated beyond the submucosa [6,7], indicating the disease at middle or late stage. At this time, the patients have already missed the optimal timing for radical surgery. Moreover, the adverse reactions resulting from chemotherapy can seriously impact the life quality of the patients, rendering the condition unfavorable for the improvement of prognosis. Therefore, it is imperative to seek novel therapeutic strategies to enhance the treatment efficacy and increase the overall survival.

Proteasome 26S non-ATPase subunit 7 (PSMD7) is a 19S regulator subunit independent of ATP that, in recent years, has been found to be overexpressed in many types of cancer cells [8]. It has been shown that in esophageal squamous cell carcinoma (ESCC), downregulated PSMD7 induces apoptosis and inhibits the aggressive behaviors of ESCC cells through the mTOR/p70S6K pathway [9]. Forkhead box P3 (FOXP3), a transcription factor involved in the carcinogenic effect of many genes and pathways including Exonuclease 1 (EXO1) in hepatocellular carcinoma, and the Wnt/β-catenin signaling pathway and Glioma-associated oncogene homolog 1 (GLI1) in non-small cell lung cancer [10–12], may bind to PSMD7 promoter according to the analysis by JASPAR database. More importantly, the activity of FOXP3 has been shown to be associated with gastric cancer cell invasion and proliferation [13,14].

In this study, we aimed to explore the role of PSMD7 in gastric cancer cells and the combination between PSMD7 and FOXP3. We hypothesized that the transcriptional activation of PSMD7 by FOXP3 may inhibit cell proliferation and facilitate cell apoptosis in gastric cancer.

## Materials and methods

### Bioinformatic tools

UALCAN database (http://ualcan.path.uab.edu/) was used for analyses of the expression of PSMD7 in normal and primary tumor tissues as well as the correlation between PSMD7 expression and cancer stages. GEPIA database (http://gepia.cancer-pku.cn/) was searched to look at the correlation between PSMD7 expression and the overall survival or the disease-free survival of gastric cancer. Cutoff high value and low value is set to 50%. P < 0.05 were considered as statistically significant using log-rank test. ROMO virtual laboratory (http://alggen.lsi.upc.es/cgi-bin/promo_v3/promo/promoinit.cgi?dirDB=TF_8.3) [15,16] predicted the transcription factors binding to PSMD7 promoter. JASPAR database (http://jaspar.genereg.net/) provided the binding sites of FOXP3 on PSMD7 promoter.

### Tissue specimens

Tumor tissues and para-carcinoma tissues were acquired from 15 patients (aged 34 to 79) diagnosed with gastric cancer and hospitalized in Haikou People’s Hospital from June 2020 to February 2021. Experiments of this study were approved by the Medical Ethics Committee of Haikou People’s Hospital. Informed consent was signed by all patients with the agreement on the use of the specimens for experimental purposes.

### Cell lines

Human gastric mucosa cell line GES-1and human gastric cancer cell lines MKN-45, MKN-74, AGS and HGC-27 (Cell Center of Shanghai Institutes for Biological Sciences) were grown in RPMI-1640 medium (Gibco) supplemented with 10% FBS and 1% penicillin/streptomycin in a humidified incubator with 5% CO_2_ at 37°C.

### Cell transfection

Small interfering RNA (siRNA) plasmids targeting PSMD7 (si-PSMD7-1 and si-PSMD7-2), empty siRNA plasmid (si-NC), FOXP3-overexpressing vector (Ov-FOXP3), empty overexpression vector (Vector), wild-type PSMD7 (WT-PSMD7) and mutant PSMD7 (MT-PSMD7) were constructed by GenePharma (Shanghai, CN). Cell transfection for different groups was performed using Lipofectamine 2000 in accordance with the protocols [17].

### Western blot

Total protein of each group was extracted by lysis buffer and quantified by BCA method. After electrophoresis of 100 to 125 μg of protein on 10% SDS-Page gel, the protein sample was transferred to polyvinylidene difluoride (PVDF) membrane blocked with 5% skimmed milk in TBST. Primary antibodies (PSMD7, 1:1000, ab178417; PCNA, 1:1000, ab29; Ki-67, 1:1000, ab92742; Bcl-2, 1:1000, ab32124; Bax, 1:1000, ab32503; cleaved caspase 3, 1:500, ab32042; caspase 3, 1:2000, ab184787; FOXP3, 1:1000, ab215206; GAPDH, 1:1000, ab8245. All from Abcam) were incubated at room temperature for 1.5 h. Secondary antibody (1:12,000, Jackson Immuno Research) was then added and incubated at room temperature for 30 min. The protein bands were visualized by using an ECL detection system (Beyotime Institute of Biotechnology, China) according to the manufacturer’s instructions [18].

### RT-qPCR

Total RNA of each group was extracted by Trizol method (Invitrogen). The cDNA was obtained by reverse transcription according to the instructions of the reverse transcription kit (Sangon, Shanghai, CN) and amplified by a PCR instrument. 3 μL of PCR product was taken to be detected through 2% non-denatured agarose gel electrophoresis. The images were analyzed by Quantity One software. The mRNA expression level was presented as the ratio of the gray level of target band to that of GAPDH [19].

### Cell counting kit-8 (CCK-8) assay

Cells of each group were seeded into 96-well plates with 3 × 10^3^ cells per well. The plates were then kept in an incubator with 5% CO2 at 37°C in saturated humidity. After incubation for 0 h, 24 h, 48 h, 72 h and 96 h, 10 μl of CCK-8 solution (Solarbio, Beijing, CN) was added to each well. Subsequently, the cells were incubated in a CO2 incubator at 37°C for 1 h. The absorbance of each well (A450 value) was measured by a spectrophotometer [20].

### Colony formation assay

Cells were seeded into a 6-well plate (5 × 10^2^ cells/well) and were allowed to grow for 10 days in complete culture medium. After the plate was washed with PBS, cells were fixed by formalin and stained with crystal violate (Aladdin, Shanghai, China) for observation and photography purposes.

### Terminal deoxynucleotidyl transferasemediated dUTP nick end-labeling (TUNEL) assay

Elabscience® One-step TUNEL Assay Kit (Green, AF488) was used to observe apoptotic gastric cancer cells [21]. DNA strand breaks of cells treated with DNAse I in different groups showed intense green fluorescent. The cells were then counterstained with DAPI (blue).

### Dual-luciferase reporter assay

For verification of the binding affinity of FOXP3 to PSMD7 promoter, cells were transfected with the luciferase reporter vectors (Vector/Ov-FOXP3 + WT/MT-PSMD7), incubated for 48 h and centrifuged for collection of cell supernatant. The ratio of the activity of firefly luciferase to that of Renilla luciferase was seen as the relative luciferase activity [22].

### Chromatin immunoprecipitation (ChIP)

Magna chip^TM^ ChIP kit (Millipore) was used for ChIP assay [23]. Chromatin was isolated, and the cells were crushed by Sonics VC750. ChIP was carried out after nonspecific antibodies were removed from the chromatin. The protein /DNA complex was eluted and de-crosslinked, followed by DNA purification. PSMD7 expression was detected by PCR.

### Statistical analysis

SPSS 13.0 software was used for statistical processing of the data, and the results were expressed as the mean ± standard deviation. One-way ANOVA and student’s t test were used for comparison between groups. P < 0.05 indicates that the difference is significant.

## Results

In this study, we explored the biological role of PSMD7 and the potential mechanism in gastric cancer cells. The data revealed that the expression of PSMD7 was significantly increased in gastric cancer tissues and cell lines. The silencing of PSMD7 suppressed cell proliferation and promoted cell apoptosis in HGC-27 cells. In addition, PSMD7 was activated by FOXP3 transcriptionally. FOXP3 overexpression increased cell viability and repressed apoptosis in HGC-27 cells. However, the interference with PSMD7 reversed the effects of FOXP3 overexpression on HGC-27 cells.

### Upregulated PSMD7 expression level in gastric cancer tissues and cell lines

**Figure 1.**
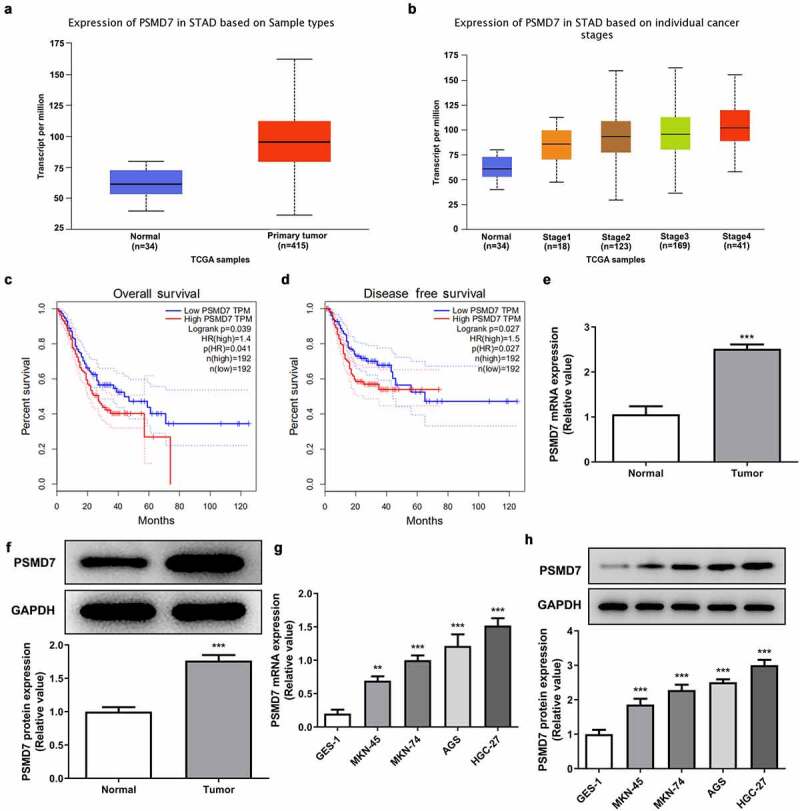
**Upregulated PSMD7 expression level in gastric cancer tissues and cell lines**. (a) Expression of PSMD7 in stomach adenocarcinoma (STAD) based on sample types, from UALCAN. (b) Expression of PSMD7 in STAD based on individual cancer stages, from UALCAN. (c) The correlation between PSMD7 expression and the overall survival of gastric cancer, analyzed by GEPIA. (d) The correlation between PSMD7 expression and the disease free survival of gastric cancer, analyzed by GEPIA. The mRNA (e) and protein expression (f) of PSMD7 in gastric cancer tissues and adjacent normal tissues, detected by Western blot. ***P < 0.001 vs Normal. (G&H) PSMD7 expression in human gastric mucosa cell line GES-1and human gastric cancer cell lines MKN-45, MKN-74, AGS and HGC-27, detected by qPCR and Western blot. **P < 0.01, ***P < 0.001 vs GES-1.

Data from UALCAN showed that the expression of PSMD7 in gastric cancer primary tumor samples is significantly higher than in normal sample, and that PSMD7 expression level is positively correlated with cancer stages (Figure 1(a-b)). The results shown on GEPIA database illustrated a close association between high PSMD7 level and lower overall survival as well as lower disease free survival (Figure 1(c-d)). Consistently, much higher level of PSMD7 mRNA and protein expression was detected by RT-qPCR and Western blot in the cancerous tissues from gastric cancer patients (Figure 1(e-f)). Gastric cancer cell lines MKN-45, MKN-74, AGS and HGC-27 all exhibited upregulated PSMD7 levels compared to GES-1, among which HGC-27 expressed the highest level of PSMD7 and was thus selected for following experiments (Figure 1(g-h)).

### PSMD7 silencing inhibited gastric cancer cell proliferation and colony formation

**Figure 2.**
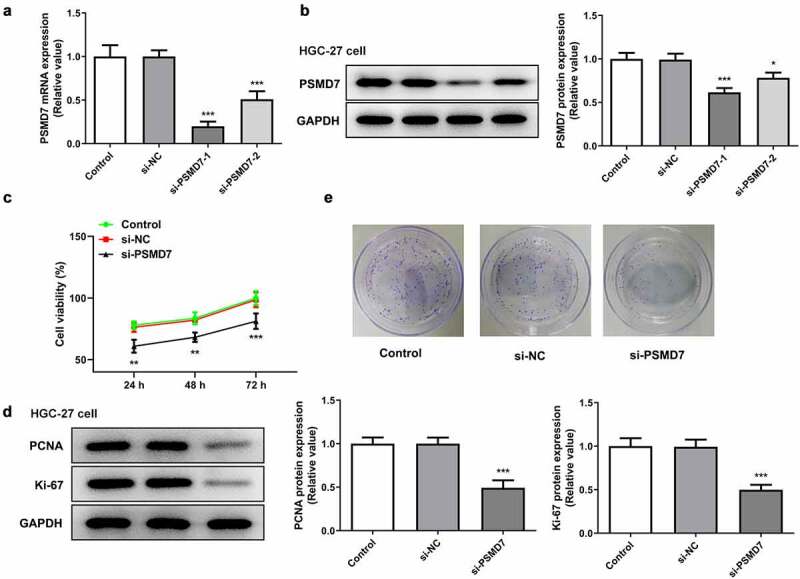
**PSMD7 silencing inhibited gastric cancer cell proliferation and colony formation**. (A&B) Results of qPCR and Western blot established the successful knockdown of PSMD7 by the transfection of PSMD7 siRNA plasmids. *P < 0.05, ***P < 0.001 vs si-NC (c) The viability of cells transfected with none, si-NC or si-PSMD7, detected by CCK-8 assay. **P < 0.01, ***P < 0.001 vs si-NC (d) The expression of proliferation markers PCNA and Ki-67 after transfection of si-PSMD7 in HGC-27 cells, detected by Western blot. ***P < 0.001 vs si-NC (e) The colonies of cells transfected with none, si-NC or si-PSMD7, detected by colony formation assay.

The expression of PSMD7 was knocked down by siRNA plasmid interference to carry out loss-of-function experiments on the role of PSMD7 in gastric cancer (Figure 2(a-b)). The expression of PSMD7 was lower in HGC-27 cells transfected with si-PSMD7-1 than in those transfected with si-PSMD7-2. Therefore, si-PSMD7-1 was selected for the rest of the experiments. Furthermore, the viability rate of cells transfected with si-PSMD7-1 was found to be greatly lower than that of cells transfected with si-NC (Figure 2(c)). The protein expression of proliferation markers PCNA and Ki-67 decreased with the downregulation of PSMD7 expression (Figure 2(d)). Gene interference with PSMD7 also reduced the number of cell colonies, by contrast with the negative control (Figure 2(e)). Therefore, these results demonstrated that PSMD7 silencing inhibited gastric cancer cell proliferation and colony formation.

### PSMD7 silencing expedited gastric cancer cell apoptosis

**Figure 3.**
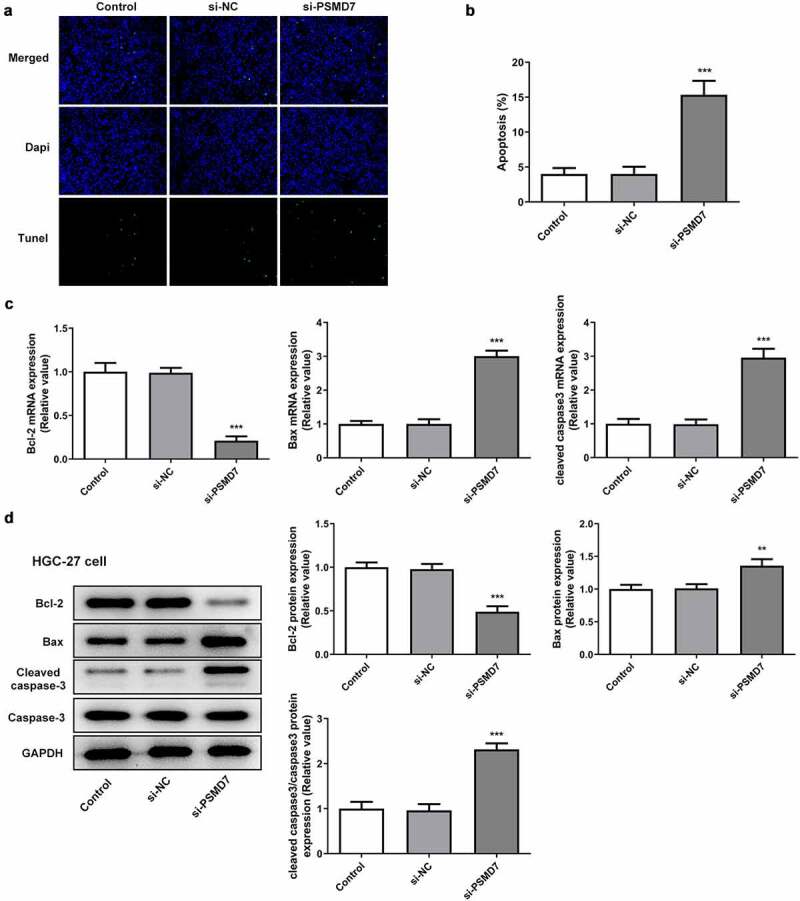
**PSMD7 silencing expedited gastric cancer cell apoptosis**. (A&B) Apoptotic HGC-27 cells after transfection of none, si-NC or si-PSMD7, and the apoptosis rate, detected through TUNEL assay. ***P < 0.001 vs si-NC (C&D) The expression of Bcl-2, Bax and cleaved caspase 3 in cells transfected with si-PSMD7, detected by qPCR and Western blot. **P < 0.01, ***P < 0.001 vs si-NC.

It can be seen from Figure 3(a) that compared to the negative control, the number of TUNEL-positive cells increased after PSMD7 silencing, suggesting increased apoptosis rate (Figure 3(b)). Moreover, HGC-27 cells transfected with si-PSMD7-1 showed a decrease in the expression of anti-apoptotic Bcl-2 and an increase in the expression of apoptosis markers Bax and cleaved caspase 3 (Figure 3(c-d)). Thus, PSMD7 silencing expedited the apoptosis of HGC-27 gastric cancer cells.

### Transcriptional activation of PSMD7 by FOXP3

**Figure 4.**
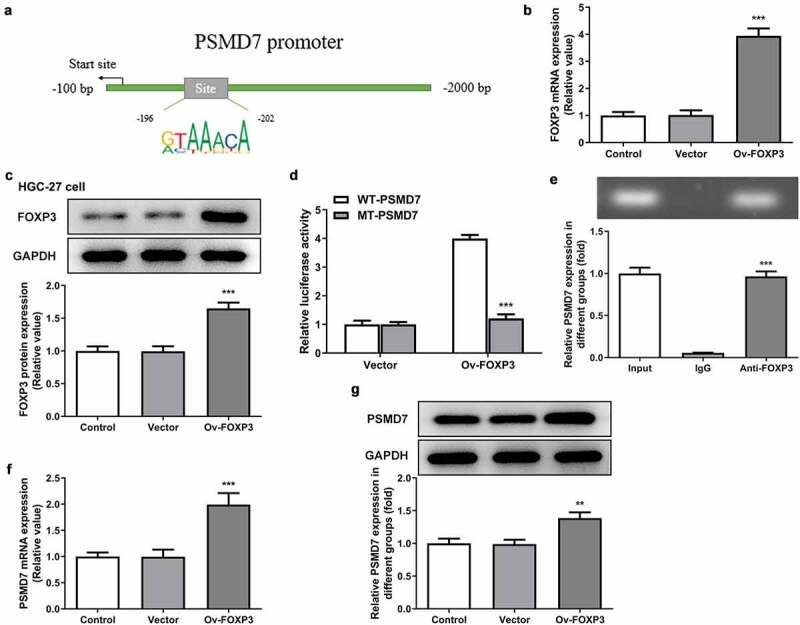
**Transcriptional activation of PSMD7 by FOXP3**. (a) FOXP3 binding sites on PSMD7 promoter, predicted by JASPAR. (B&C) FOXP3 expression after transfection of Ov-FOXP3 in HGC-27 cells, detected by qPCR and Western blot. ***P < 0.001 vs Vector (d) Luciferase activity in cells transfected with Vector/Ov-FOXP3 and WT/MT-PSMD7, detected by dual-luciferase reporter assay. (e) PSMD7 enrichment after immunoprecipitation with Anti-FOXP3 or IgG, detected by ChIP assay. ***P < 0.001 vs IgG (F&G) PSMD7 expression in cells transfected with none, Vector or Ov-FOXP3, detected by qPCR and Western blot. **P < 0.01, ***P < 0.001 vs Vector.

PROMO database is identified FOXP3 as a transcription factor predicted to bind to PSMD7 promoter with a dissimilarity margin less or equal than 0%. The binding sites of FOXP3 on PSMD7 promoter sequences was analyzed by JASPAR database (Figure 4(a)). The results of qPCR and Western blot established the successful overexpression of FOXP3 in HGC-27 cells (Figure 4(b-c)). Moreover, cells transfected with Ov-FOXP3 and WT-PSMD7 showed a conspicuously higher level of luciferase activity than cells transfected with Ov-FOXP3 and MT-PSMD7, while the Vector group showed no noticeable changes (Figure 4(d)). The result of ChIP assay demonstrated noticeably enriched PSMD7 in the Anti-FOXP3 group, compared to the IgG group (Figure 4(e)). In addition, PSMD7 upregulation was observed after FOXP3 overexpression in HGC-27 cells (Figure 4(f-g)). These results collectively illustrated the transcriptional activation of PSMD7 by FOXP3.

### PSMD7 silencing reversed FOXP3 overexpression-promoted gastric cancer cell proliferation and colony formation

**Figure 5.**
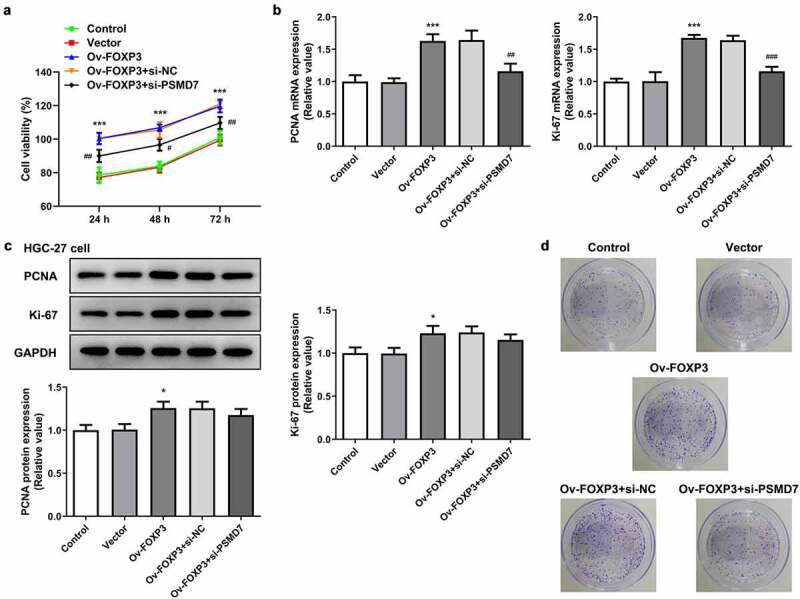
**PSMD7 silencing reversed FOXP3 overexpression-promoted gastric cancer cell proliferation and colony formation**. (a) The viability of cells transfected with none, Vector, Ov-FOXP3, Ov-FOXP3+ si-NC, Ov-FOXP3+ si-PSMD7, detected by CCK-8 assay. ***P < 0.001 vs Vector; ^#^P < 0.05, ^##^P < 0.01 vs Ov-FOXP3+ si-NC (B&C) The expression of PCNA and Ki-67 in cells transfected with none, Vector, Ov-FOXP3, Ov-FOXP3+ si-NC, Ov-FOXP3+ si-PSMD7, detected by qPCR and Western blot. *P < 0.05, ***P < 0.001 vs Vector; ^##^P < 0.01 vs Ov-FOXP3+ si-NC (d) Colonies of cells transfected with none, Vector, Ov-FOXP3, Ov-FOXP3+ si-NC, Ov-FOXP3+ si-PSMD7, observed through colony formation assay.

HGC-27 cells were also transfected with Ov-FOXP3 to perform gain-of-function experiments on the role of FOXP3 in gastric cancer. It was found that FOXP3 overexpression resulted in an overall higher level of cell viability, in comparison with the Vector group (Figure 5(a)). Co-transfection of Ov-FOXP3 and si-PSMD7, however, brought down the viability rate compared to the si-NC group. Similar results were observed in terms of the expression of proliferation markers, as FOXP3 overexpression elevated the expression levels of PCNA and Ki-67, while the presence of si-PSMD7 reversed this effect (Figure 5(b-c)). Additionally, more cell colonies were seen in the Ov-FOXP3 group than the Vector group, whereas co-transfection of Ov-FOXP3 and si-PSMD7 decreased cell colony formation capacity compared to the si-NC group (Figure 5(d)). These results indicated that PSMD7 silencing reversed FOXP3 overexpression-promoted gastric cancer cell proliferation and colony formation.

### PSMD7 silencing reversed FOXP3 overexpression-inhibited gastric cancer cell apoptosis

**Figure 6.**
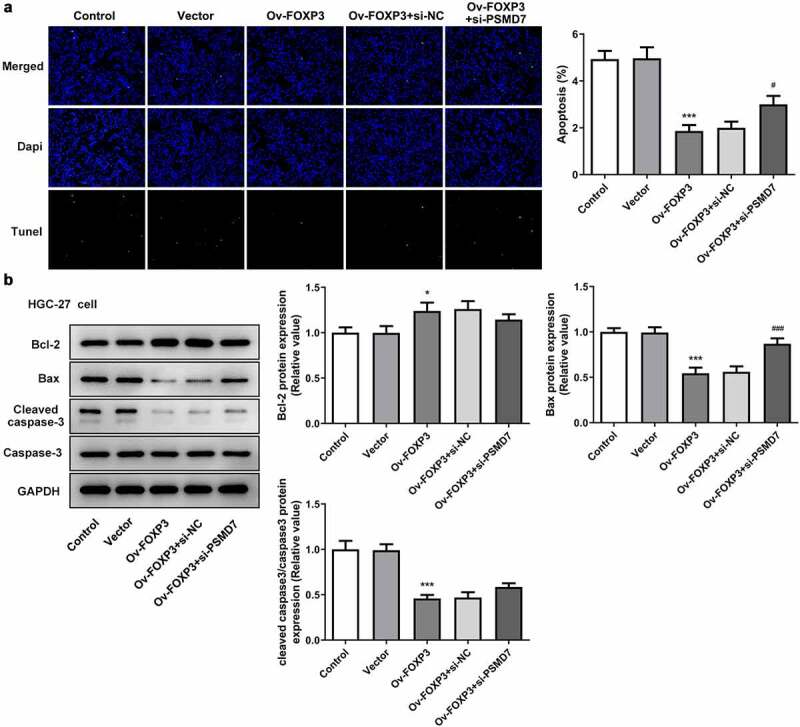
**PSMD7 silencing reversed FOXP3 overexpression-inhibited gastric cancer cell apoptosis**. (a) Apoptotic cells and cell apoptosis rate in HGC-27 transfected with none, Vector, Ov-FOXP3, Ov-FOXP3+ si-NC, Ov-FOXP3+ si-PSMD7, detected by TUNEL. ***P < 0.001 vs Vector; ^#^P < 0.05 vs Ov-FOXP3+ si-NC (b) The expression of Bcl-2, Bax and cleaved caspase 3 in cells transfected with none, Vector, Ov-FOXP3, Ov-FOXP3+ si-NC, Ov-FOXP3+ si-PSMD7, detected by Western blot. *P < 0.05, ***P < 0.001 vs Vector; ^###^P < 0.01 vs Ov-FOXP3+ si-NC.

It was observed from TUNEL assay that the apoptosis rate of HGC-27 cells decreased after the overexpression of FOXP3, whereas interference with PSMD7 offset this effect of FOXP3 overexpression (Figure 6(a)). The results of Western blot further verified this finding, as the FOXP3 overexpression group showed Bcl-2 upregulation and a downregulation of Bax and cleaved caspase 3, and the Ov-FOXP3 + si-PSMD7 group showed the opposite trends (Figure 6(b)). Thus, PSMD7 silencing reversed FOXP3 overexpression-inhibited gastric cancer cell apoptosis.

## Discussion

Gastric cancer is one of the most common digestive system neoplasms and is about the third leading cause of cancer-related deaths worldwide [24]. The incidence of gastric cancer has great regional differences and is especially high in Eastern Asia [25,26]. Consumption of foods high in salt or nitrate, alcohol consumption and cigarette smoking have been shown to be major risk factors for gastric cancer [27]. In addition to genetic susceptibility, a family history of gastric cancer is also a risk factor [28]. The early development of gastric cancer is, in most cases, asymptomatic and thus easily ignored. Therefore, patients with gastric cancer tend to be diagnosed in advanced stages accompanied by distant metastasis, with low cure rate, easy recurrence and poor prognosis [29]. Therefore, it is an important task for medical practitioners and scientific researchers to search for meaningful biological indicators of gastric cancer and molecular targets that further support current targeted therapy and neoadjuvant chemoradiotherapy [30].

Through GEO2R analysis of the GSE118916 chip expression data from the National Center for Biotechnology Information (NCBI), it was found that PSMD7 is highly expressed in pancreatic cancer tissues. According to the results from UALCAN database, the expression level of this gene in stomach adenocarcinoma tissues was significantly higher than that in normal gastric tissues. Meanwhile, GEPIA database analyzed the correlation between PSMD7 expression and the disease survival and prognosis and reported that the overall and disease-free survival rates of patients with low PSMD7 expression was significantly higher than those of patients with high PSMD7 expression. Therefore, PSMD7 promoting gastric cancer tumorigenesis could be a reasonable conjecture. A previous study focused on esophageal squamous cell carcinoma has reported that downregulating PSMD7 expression level could effectively elicit the apoptosis and suppress the proliferation of the cancer cells [9]. Another study has also observed accelerated apoptosis and inhibited tumor growth of lung adenocarcinoma after the knockdown of PSMD7 in vitro and in vivo [31]. The present determined PSMD7 upregulation in both gastric cancer tissues and cell lines. Additionally, silencing PSMD7 in gastric cancer cell line HGC-27 was shown to be able to induce the loss of cell viability and proliferative capacity and speed up cell apoptosis.

In order to understand the mechanism responsible for the function of PSMD7 in gastric cancer cell proliferation and apoptosis, bioinformatic tools including PROMO and JASPAR databases were searched for potential interaction between PSMD7 and putative transcription factors. FOXP3 was found to have binding sites on PSMD7 promoter and its role as a transcriptional activator of PSMD7 was corroborated in the present study. Previous researches have supported the positive correlation between the transcription and expression of FOXP3 and the lesion grade of gastric cancer [32,33]. Elevated FOXP3 expression has also been found in the gastric tumor specimens and lymph nodes from patients with metastatic gastric cancer [34]. FOXP3 has proven to participate in the aggressive activities of gastric cancer cells such as proliferation, migration and invasion via the TGF-β signaling pathway [35]. The overexpression of FOXP3 in the present study led to noticeably increased viability and proliferation of HGC-27 cells and decreased cell apoptosis, whereas interference with PSMD7 could effectively reduce these effects of FOXP3 overexpression.

However, there are several limitations in this study. Firstly, we explore the role of FOXP3-mediated PSMD7 in proliferation and apoptosis in gastric cancer cells, but more functional experiments should be assessed to widely investigate the role of PSMD7 in gastric cancer. Secondly, we only performed the experiments at cell level, and we will verify the results in animal models in further study. Thirdly, the tumor microenvironment is heterogeneous and is comprised of many cell types. We used RT-qPCR and Western blot assay to quantify the PSMD7 expression in gastric cancer tissues, but we did not explore the expression pattern of PSMD7. Clinical immunohistochemical assay may be useful to locate PSMD7 expression in gastric cancer tissues. Fourthly, as far we as we know, PSMD7 is overexpressed in many cancer cells, indicating the tumor-promoting role of PSMD7. When we silenced PSMD7, the viability of cancer cells was inhibited. It is worth noting that PSMD7 is required for general cell survival, and whether the reduced cell viability is specific to gastric cancer cells is still unclear. Thus, it is necessary to continue investigating the effects of PSMD7 silencing on other cell types. Finally, the special mechanisms underlying the cellular effects of PSMD7 knockdown on gastric cancer cells are still unclear, which will be our focus in further study.

## Conclusion

In conclusion, this study established that PSMD7 could be transcriptionally activated by FOXP3 in HGC-27 cells and facilitate cell proliferation and inhibit cell apoptosis in gastric cancer. PSMD7 and FOXP3 have the potential to be meaningful biological indicators of gastric cancer and may be applied to the development of novel agents or targeted therapy as candidate molecular targets.

## Highlights

- The silencing of PSMD7 inhibits proliferation and induces apoptosis in gastric cancer cells.
- FOXP3 transcriptionally activates PSMD7.
- FOXP3 promotes the proliferation and inhibits apoptosis in gastric cancer cells by binding to the PSMD7 promoter.
